# Standardization of the face-hand test in a Brazilian multicultural population: prevalence of sensory extinction and implications for neurological diagnosis

**DOI:** 10.6061/clinics/2016(12)08

**Published:** 2016-12

**Authors:** Gustavo José Luvizutto, Marcelo Ortolani Fogaroli, Rodolfo Mazeto Theotonio, Hélio Rubens de Carvalho Nunes, Luiz Antônio de Lima Resende, Rodrigo Bazan

**Affiliations:** I Universidade Estadual Paulista – UNESP, Faculdade de Medicina de Botucatu, Neuro reabilitação, Botucatu/SP, Brazil; II Universidade Estadual Paulista – UNESP, Faculdade de Medicina de Botucatu, Estudante de Medicina, Botucatu/SP, Brazil; III Universidade Estadual Paulista – UNESP, Faculdade de Medicina de Botucatu, Departamento de Saúde Pública, Botucatu/SP, Brazil; IV Universidade Estadual Paulista – UNESP, Faculdade de Medicina de Botucatu, Departamento Neurologia, Psicologia e Psiquiatria, Botucatu/SP, Brazil

**Keywords:** Face-hand Test, Diagnosis, Psychiatric Syndromes, Unilateral Spatial Neglect

## Abstract

**OBJECTIVE:**

The face-hand test is a simple, practical, and rapid test to detect neurological syndromes. However, it has not previously been assessed in a Brazilian sample; therefore, the objective of the present study was to standardize the face-hand test for use in the multi-cultural population of Brazil and identify the sociodemographic factors affecting the results.

**METHODS:**

This was a cross sectional study of 150 individuals. The sociodemographic variables that were collected included age, gender, race, body mass index and years of education. Standardization of the face-hand test occurred in 2 rounds of 10 sensory stimuli, with the participant seated to support the trunk and their vision obstructed in a sound-controlled environment. The face-hand test was conducted by applying 2 rounds of 10 sensory stimuli that were applied to the face and hand simultaneously. The associations between the face-hand test and sociodemographic variables were analyzed using Mann-Whitney tests and Spearman correlations. Binomial models were adjusted for the number of face-hand test variations, and ROC curves evaluated sensitivity and specificity of sensory extinction.

**RESULTS:**

There was no significant relationship between the sociodemographic variables and the number of stimuli perceived for the face-hand test. There was a high relative frequency of detection, 8 out of 10 stimuli, in this population. Sensory extinction was 25.3%, which increased with increasing age (OR=1.4[1:01–1:07]; *p*=0.006) and decreased significantly with increasing education (OR=0.82[0.71-0.94]; *p*=0.005).

**CONCLUSION:**

In the Brazilian population, a normal face-hand test score ranges between 8–10 stimuli, and the results indicate that sensory extinction is associated with increased age and lower levels of education.

## INTRODUCTION

Bender et al. developed the face-hand test (FHT) to investigate specific patterns of neurological disorders through dual concurrent sensory stimulation of the face and back of the hand 1. They found that the FHT had 2 response patterns: sensory extinction (only one stimulus is recognized by the individual) or displacement (stimuli are recognized elsewhere in the body). Based on these findings, the FHT was proposed in Brazil by Blay et al. as a tool for assessing patients with psychiatric and neurologic diseases 2,3.

The first standardization of the FHT occurred in 1969, based on the results from 3 groups of volunteers who were categorized by age (3–6 years old, 7–12 years, and older than 12 years). The results of the FHT in these 3 groups were compared to the FHT scores of patients with schizophrenia, organic psychosis, or aphasia. The authors concluded that the most common errors for sensory extinction occurred predominantly in the face of patients with organic psychosis and children who were 3–6 years old 4-5.

The FHT has also been used to diagnose perceptual neurological syndromes, such as for clinical differential diagnosis of unilateral spatial neglect (USN). Feinberg et al. evaluated the FHT scores of patients with unilateral hemispheric lesions 3 months after stroke and found both contralateral and ipsilateral USN in patients with right hemisphere lesions. However, similar findings did not occur in patients with lesions to the left hemisphere 6. These findings are important for explaining ipsilateral extinction and indicate a role of the right hemisphere in the mechanisms of spatial attention. These findings also support that the test is precise enough to detect changes in the perceptions of individuals with neurological conditions 7-10.

The FHT is a simple test, as well as being practical and fast, with a high sensitivity to detect psychiatric syndromes and USN after stroke. However, the FHT has not previously been assessed in a Brazilian sample; therefore, the objective of the present study was to standardize the FHT for use in the multi-cultural population of Brazil as well as to identify the main sociodemographic factors affecting the test results. The central hypothesis was that sensory stimuli scores of approximately 10 are typical in the population and that abnormal patterns on the FHT, such as sensory extinction and displacement, may be associated with lower education levels.

## PATIENTS AND METHODS

### Participants

This cross-sectional study included graduate students in the Botucatu Medical School (UNESP) as well as professionals and patients at the Clinic Hospital of Botucatu, who were recruited through direct contact with the researcher followed by invitation to participate in the study.

**Figure 1:**
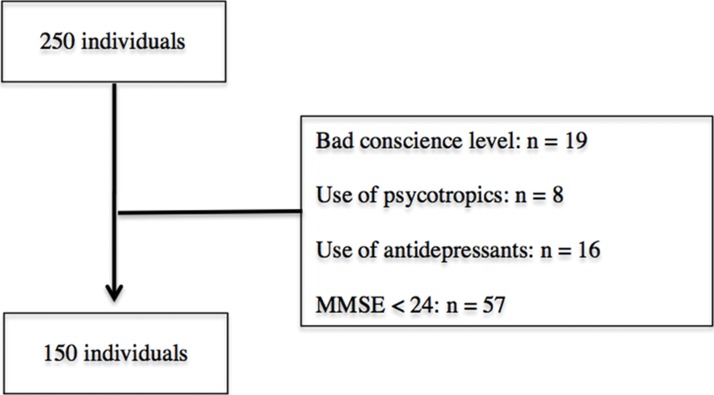
The screening process, indicating patients excluded from the study.

Participants met the following inclusion criteria (Figure 1): no history of neurological disease in the central nervous system or peripheral nervous system, such as peripheral neuropathy, alcohol abuse, or hypothyroidism; participants also had to be conscious during testing, could not be currently taking psychotropic drugs or antidepressants, and had to have a score>24 on the Mini-Exam Mental State Examination (MMSE). The MMSE cutoff score was selected to correspond with the most commonly used value in clinical and epidemiological studies of dementia in Brazil 11.

### Study variables

The sociodemographic data obtained during interviews with patients were as follows: age (years), gender (men and women), race (Caucasian and non-Caucasian), body mass index (BMI, kg/m^2^), and years of education.

Standardization of the FHT was performed with the participant seated to support the trunk, with their vision obstructed, in a sound-controlled environment. The FHT was conducted by applying 2 rounds of 10 sensory stimuli with cotton in a craniocaudal direction applied at dorsum of the hand at the 3rd metacarpal. This was followed by 10 stimuli applied to the cheek region of the face and 10 simultaneous stimuli applied to the face and hand. All stimuli were initially applied on the left side and then applied on the right. Finally, the number of rings (sensory stimuli) and the location of touch perceived by the individual in each testing segment were categorized as either normal sensory extinction or displacement. Table 1 shows the sequence of stimuli applied during the FHT.

### Statistical analyses

Since we are using a sample representative of the target population, our sampling is considered to be intentional and non-probabilistic. We needed a minimum of 150 subjects to obtain a maximum sampling error of 7.5% and a confidence level of 95%. The associations between FHT scores and the sociodemographic variables gender and race were analyzed using nonparametric Mann-Whitney tests. The associations between FHT scores and age, BMI, and years of education were assessed using Spearman correlations. An adaptation of the binomial distribution assumption was used to model the perceived number of touches, followed by calculating the maximum likelihood estimates for the binomial distribution parameters for each variation of the FHT. The relationships between sociodemographic variables and sensory extinction on the FHT were analyzed by multiple logistic regression, adjusted for age and years of education. After adjustments, receiver operating characteristic (ROC) curves were calculated for age and years of education to establish values that maximize the sensitivity and specificity of sensory extinction on the FHT. Associations were considered statistically significant if *p*<0.05. Analyses were performed using SPSS software (version 21.0, SPSS, Chicago, IL, USA).

### Ethics

The study was approved by the Ethics in Human Research Committee under protocol 4223/2012, and all participants gave written informed consent.

## RESULTS

We evaluated and screened 250 individuals, but only 150 participants met the inclusion criteria for the study (Figure 1). The sociodemographic characteristics of the participants are presented in Table 2. Tables 3 and 4 demonstrate that there were no significant associations between FHT scores and sociodemographic variables, including gender, race, age, BMI, and years of education.

Table 5 presents data for the number of touches perceived, according to FHT variations. We observed a relatively high percentage of participants who perceived at least 8 touches during all variations of the FHT, particularly during the face right (Fr), face left (Fl), face-hand right (FH-r), and FH variations, in which more than 90% of participants noticed at least 8 touches. The percentage of participants perceiving 8 or more touches was lower for the hand right (Hr) and H variations, but was still >80%.

Table 6 shows the binomial models adjusted for the number of rings perceived in each variation of the FHT.

Table 7 shows the association between sociodemographic variables and the probability of sensory extinction during the FHT. We observed a statistically significant increase in the probability of sensory extinction with increasing age (OR=1.04, range 1.01–1.07; *p*=0.006), and a significant reduction in the probability of extinction with increasing years of education (OR=0.82, range 0.71–0.94; *p*=0.005).

**Figure 2:**
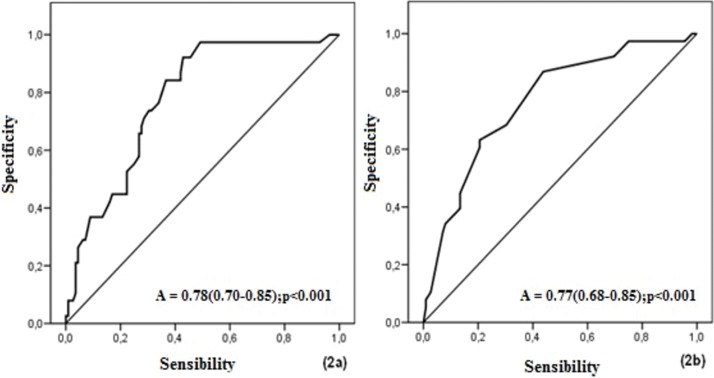
Receiver operating characteristics (ROC) curves for age (2a) and years of education (2b) to determine the values that maximize the sensitivity and specificity of sensory extinction in the face-hand test (FHT).

Figure 2 shows the ROC curves for effects of age and years of education, which were used to establish values that maximize the sensitivity and specificity for sensory extinction detected by the FHT. For age, a sensitivity and specificity of 68.4% and 72.3%, respectively, and 41.5 years of age produced an area under the curve of 0.78 (95% CI=0.70–0.85; *p*<0.001; Figure 2a). For years of education, a sensitivity and specificity of 68.4% and 69.6%, respectively, were associated with 10.5 years and generated an area under the curve of 0.77 (95% CI; 0.68–0.85; *p*<0.001; Figure 2b).

## DISCUSSION

The present study accomplished standardization of the FHT in a typical population without neurological disorders and demonstrated low variability for <8 touch stimuli, with the highest frequency of stimulation between 8 and 10. The initial normative study suggests that no stimuli should be neglected, that the most common error resulted from sensory extinction by the stimulus and that responses are less accurate in patients with organic psychological syndromes and in children 3–6 years of age with face dominance 1.

We found associations between sensory extinction and increasing age as well as lower education. The number of years of education is associated with neuropsychological performance on tasks that assess various brain functions, such as memory, attention, language, and executive functions. In studies on regulation, or in comparative analyses between groups, education is often the most relevant variable, followed or accompanied by age 12. Additional education may be associated with increased synaptic connections or cerebral vasculature, thereby increasing higher cortical functioning 13-14.

We observed that 25.3% of participants had sensory extinction during double stimulation of the face and hand. Under the original classification system, the FHT is divided into 4 distinct groups: (A) individuals who detect all applied stimuli, (B) individuals who detect sensory stimuli only in the face, (C) individuals who detect 2 simultaneous sensory stimuli in the face, and (D) individuals who detect sensory stimuli only in the hands. Previous studies have demonstrated that most errors were in relation to extinction in the face 5. In a study that found an association between EEG activity and sensory stimulation of the median or tibial nerves, it demonstrated cortical dominance of hand functions 8.

Another potential application of the FHT is for evaluation of the attention network, comprising the right perisylvian region (posterior parietal lobe, superior temporal cortex, as well as middle and prefrontal cortex). The FHT may be used to test errors that influence this network, with a potential application for the diagnosis of syndromes such as unilateral spatial neglect 15-19. The FHT may also demonstrate that patients with right hemisphere USN present with both ipsilateral and contralateral sensory deficits, whereas patients with left hemisphere damage present with deficits on only the right side, indicating right hemisphere dominance for attention and somatosensory integration 20-21.

The limitations of the present study include recruitment of participants through a single research center and the absence of a comparison of the findings with participants with psychological or organic diseases. However, we demonstrated normal score ranges and outlined benchmarks for their application to clinical practice. Another limitation relates to the intensity of the stimulus applied, as stimulus intensity can directly interfere with sensation. In our study, the stimulus intensity could not be measured objectively, but we did have the same researcher apply all stimuli to reduce the potential for confounding effects. Additionally, given that factors such as nociceptive processes, respiratory discomfort, or other sensations may interfere with stimulus perception, we conducted the tests in a controlled stimulation room with minimal external environmental stimuli.

In conclusion, in a Brazilian multicultural population, normal responses for the FHT, which presents patterns of simultaneous stimulation, are scores between 8 and 10. Additionally, sensory extinction is associated with increased age, with a cutoff point of 41 years. Sensory extinction is also associated with fewer than 5 years of education, with a cutoff point of 10.5 years.

## AUTHOR CONTRIBUTIONS

Resende LA and Bazan R designed and projected the study. Luvizutto GJ, Fogaroli MO and Theotonio RM were responsible for the production (data collection, data tabulation). Nunes HR was responsible for the statistical analyses. Luvizutto GJ and Bazan R were responsible for the manuscript writing manuscript. Luvizutto GJ, Bazan R and Resende LA were responsible for the final discussion.

## Figures and Tables

**Table 1:** Sequences of stimuli applied during the face-hand test (FHT).

1^st^ round of stimuli:
1. 10 sensory stimuli to the right hand
2. 10 sensory stimuli to the right face
3. 10 sensory stimuli to the right face and the right hand
2^nd^ round of stimuli:
4. 10 sensory stimuli to the left hand
5. 10 sensory stimuli to the left face
6. 10 sensory stimuli to the left hand and the left face

**Table 2:** Sociodemographic characteristics of the sample (n=150).

Variable	Summary
Gender (women:men)	176 (50.7%):74 (49.3%)
Race (Caucasian:non-Caucasian)	112 (74.7%):38 (25.3%)
Years of education^1^	11 (0–16)
Age^1^ (years)	31.5 (18.0–87.0)
BMI^1^ (kg/m^2^)	22.9 (11.7–35.9)
F-r^1^	10.0 (3.0–12.0)
F-l^1^	10.0 (4.0–13.0)
H-r^1^	10.0 (2.0–12.0)
H-l^1^	10.0 (3.0–13.0)
FH-r^1^	10.0 (2.0–11.0)
FH-l^1^	10.0 (3.0–13.0)
Sensorial extinction	38 (25.3%)

1 Summary median (min-max); BMI = body mass index; F-r = right face; F-l = left face; H-r = right hand; H-l = left hand; FH-r = right face-hand; FH-l = left face-hand.

**Table 3:** Face-hand test (FHT) scores by gender and race.

	Gender			Race		
FHT	Women (n=76)	Men (n=74)	p^1^	Caucasian (n=112)	Non-Caucasian (n=38)	p^1^
F-r	10(3–12)	10(6–12)	0.950	10(5–12)	10(3–12)	0.805
F-l	10(4–13)	10(5–12)	0.627	10(5–12)	10(4–13)	0.686
H-r	10(2–11)	10(3–12)	0.529	10(3–11)	10(2–12)	0.132
H-l	10(3–13)	10(3–11)	0.124	10(5–11)	10(3–13)	0.755
FH-r	10(2–11)	10(2–11)	0.887	10(2–11)	10(2–11)	0.466
FH-l	10(3–13)	10(5–12)	0.880	10(5–13)	10(3–12)	0.434

1 Mann-Whitney. Summary in median (min-max); F-r = right face; F-l = left face; H-r = right hand; H-l = left hand; FH-r = right face-hand; FH-l = left face-hand.

**Table 4:** Correlation between face-hand test (FHT) scores and age, BMI, or years of education.

Factor	F-r	F-l	H-r	H-l	FH-r	FH-l
	ρ (*p* value)	ρ (*p* value)	ρ (*p* value)	ρ (*p* value)	ρ (*p* value)	ρ (*p* value)
Age (years)	-0.29(<0.001)	0.03(0.674)	-0.35(<0.001)	-0.10(0.218)	-0.27(<0.001)	-0.21(0.009)
BMI (kg/m^2^)	0.04(0.588)	0.10(0.221)	-0.05(0.517)	-0.006(0.941)	0.03(0.628)	0.10(0.186)
Years of education	0.29(<0.001)	0.17(0.03)	0.32(<0.001)	0.34(<0.001)	0.23(0.004)	0.24(0.002)

F-r = right face; F-l = left face; H-r = right hand; H-l = left hand; FH-r = right face-hand; FH-l = left face-hand. ρ = Spearman correlation.

**Table 5:** Number of touches by face-hand test (FHT) variation.

Number of touches on FHT	FHT variation											
	F-r		F-l		H-r		H-l		FH-r		FH-l	
	n	fr	n	fr	n	fr	n	fr	n	fr	n	fr
0	0	0.000	0	0.000	0	0.000	0	0.000	0	0.000	0	0.000
1	0	0.000	0	0.000	0	0.000	0	0.000	0	0.000	0	0.000
2	0	0.000	0	0.000	1	0.007	0	0.000	2	0.013	0	0.000
3	1	0.007	0	0.000	2	0.013	2	0.013	1	0.007	1	0.007
4	1	0.007	2	0.013	2	0.013	2	0.013	0	0.000	0	0.000
5	2	0.013	1	0.007	6	0.040	4	0.027	3	0.020	3	0.020
6	6	0.040	3	0.020	3	0.020	3	0.020	3	0.020	3	0.020
7	2	0.013	4	0.027	9	0.060	6	0.040	1	0.007	3	0.020
8	7	0.047	10	0.067	8	0.053	5	0.033	7	0.047	5	0.033
9	9	0.060	13	0.087	11	0.073	19	0.127	12	0.080	13	0.087
10	122	0.813	117	0.780	108	0.720	109	0.727	121	0.807	122	0.813
≥ 8 touches	138	0.920	140	0.934	127	0.846	133	0.887	140	0.933	140	0.933

Legend: n = number of touches; f = relative frequency of perceived touches; F-r = right face; F-l = left face; H-r = right hand; H-l = left hand; FH-r = right face-hand; FH-l = left face-hand. n = absolute frequency; fr = relative frequency.

**Table 6:** Probabilistic models adjusted for the number of rings with each perceived change on the face-hand test (FHT), for a total of 10 stimuli.

FHT variation	Distribution	Pr [t ≥ 8]
F-r	Bin (10;0.949)	0.987
F-l	Bin (10;0.950)	0.988
H-r	Bin (10;0.913)	0.950
H-l	Bin (10;0.930)	0.971
FH-r	Bin (10;0.947)	0.986
FH-l	Bin (10;0.956)	0.991

Bin = binomial distribution; Pr = estimated probability of perceiving at least 8 of 10 stimuli received under the fitted distribution; F-r = right face; F-l = left face; H-r = right hand; H-l = left hand; FH-r = right face-hand; FH-l = left face-hand.

**Table 7:** Regression adjusted logistics for the probability of extinction on the face-hand test (FHT).

Variable	β	SE	Wald	p	OR	CI 95%
Men	-0.63	0.46	1.83	0.176	0.53	(0.22–1.32)
Age (years)	0.04	0.01	7.48	0.006	1.04	(1.01–1.07)
BMI (kg/m^2^)	-0.01	0.05	0.01	0.904	0.99	(0.90–1.10)
Race (non-Caucasian)	-0.47	0.53	0.76	0.382	0.63	(0.22–1.79)
Years of education	-0.20	0.07	7.98	0.005	0.82	(0.71–0.94)
Constant	-0.24	1.56	0.02	0.876	0.78	
